# Proteases in Plasma and Kidney of db/db Mice as Markers of Diabetes-Induced Nephropathy

**DOI:** 10.5402/2011/832642

**Published:** 2011-08-04

**Authors:** E. Hadler-Olsen, J.-O. Winberg, F. P. Reinholt, T. Larsen, L. Uhlin-Hansen, T. Jenssen, E. Berg, S. O. Kolset

**Affiliations:** ^1^Department of Medical Biology, University of Tromsø, 9037 Tromsø, Norway; ^2^Department of Pathology, Oslo University Hospital, Rikshospitalet, 0027 Oslo, Norway; ^3^Kidney Section, Department of Internal Medicine, Oslo University Hospital, Rikshospitalet, 0027 Oslo, Norway; ^4^Institute of Clinical Medicine, University of Tromsø, 9037 Tromsø, Norway; ^5^Department of Nutrition, Institute for Basic Medical Sciences, University of Oslo, P.O. Box 1046 Blindern, 0316 Oslo, Norway

## Abstract

Db/db mice are overweight, dyslipidemic and develop diabetic complications, relevant for similar complications in human type 2 diabetes. We have used db/db and db/+ control mice to investigate alterations in proteinase expression and activity in circulation and kidneys by SDS-PAGE zymography, electron microscopy, immunohistochemistry, Western blotting, and in situ zymography. Plasma from db/db mice contained larger amounts of serine proteinases compared to db/+ mice. Kidneys from the db/db mice had a significantly larger glomerular surface area and somewhat thicker glomerular basement membranes compared to the db/+ mice. Furthermore, kidney extracts from db/+ mice contained metalloproteinases with *M*_*r*_ of approximately 92000, compatible with MMP-9, not observed in db/db mice. These results indicate that higher levels of serine proteinases in plasma may serve as potential markers for kidney changes in db/db mice, whereas a decrease in MMP-9 in the kidney may be related to the glomerular changes.

## 1. Introduction

Patients with diabetes mellitus face several challenges with long-term health consequences. One of the most important factors to control in this disease is chronic hyperglycaemia. Several complications, such as retinopathy, peripheral neuropathy, and nephropathy are correlated to elevated HbA_1c_, an accepted measure of long-term blood glucose control [1]. In diabetic nephropathy, changes in the extracellular matrix (ECM) of the kidneys are common. The most prominent alterations are expansion of the glomerular mesangial matrix, increased thickness of the basement membrane in glomeruli and tubules, and increased glomerular surface area [2–6]. The ECM of the basement membranes is important for filtration and reabsorption, and changes in the synthesis, turnover, and assembly of this matrix will affect these processes. Increased kidney filtration and/or reduced reabsorption may give rise to proteinuria, a common symptom in diabetes. Several alterations may contribute to the development of proteinuria, such as increased synthesis and crosslinking of collagen type IV, decreased levels of proteoglycans, and changes in matrix turnover [7–10]. In db/db mice, increased urinary excretion of type IV collagen has been demonstrated [11]. Matrix turnover is dependent on several types of proteinases, including matrix metalloproteinases (MMPs) and their inhibitors [12]. MMPs have been shown to be involved in several types of diseases, ranging from rheumatoid arthritis and cancer to atherosclerosis and diabetes [13, 14]. Both MMP-2 (gelatinase A) and MMP-9 (gelatinase B) are present in normal kidney and considered to be important for ECM turnover in this organ [15]. Shifts in the expression of MMPs have been demonstrated in rats with streptozotocin-induced diabetes [16] and an imbalance between MMPs and their inhibitors shown to contribute to nephropathy [17]. Changes in MMP expression and activity in relation to kidney diseases have been discussed and reviewed previously [14, 18, 19]. 

 We have recently shown that the levels of MMP-2 and MMP-9 were higher in serum from subjects with type 1 diabetes than in controls, whereas the levels of tissue inhibitors of metalloproteinases (TIMPs) were not affected [20]. It has also been shown that MMP-2 is increased in urine [21], suggesting that MMPs may be biomarkers for kidney changes in diabetes. The use of MMPs in serum or plasma as markers for early kidney disease is potentially interesting. If altered ECM turnover contributes to proteinuria, it would be of interest to investigate in more detail whether kidney changes are reflected in alterations of MMP expression in circulation and kidney tissue.

 The db/db mouse is an animal model for obesity-related diabetes and may be used to study kidney changes in diabetes [22]. These mice are overweight, hyperglycaemic, and hyperinsulinemic and show increased kidney weight, increased glomerular mesangial matrix, and albumin excretion [23]. However, few studies have made use of the db/db mouse model to study kidney changes in diabetes in relation to possible changes in the synthesis or regulation of MMPs. Furthermore, there has been limited focus on possible plasma markers for early kidney changes in these mice, linked to parallel documentation of kidney changes. We have used db/db mice and the db/+ controls to determine the level of gelatin-degrading enzymes in plasma samples and kidney extracts. Furthermore, kidney sections were used to determine ultrastructural matrix changes by morphometry and in vivo proteinase activity by in situ gelatin zymography. 

## 2. Methods

### 2.1. Animals

11-12 weeks old db/db diabetic mice (mean body weight 49.2 g, *n* = 8) of mixed gender, as well as their nondiabetic heterozygote littermates (db/+, mean body weight 27.9 g, *n* = 12), were purchased from Harlan (Bicester, UK) or M&B (Ry, Denmark) and were housed at 23°C and 55% humidity with a 12 : 12 h light-dark cycle. The mice were treated in accordance to the guidelines on accommodation and care of animals formulated by the European Convention for the Protection of Vertebrate Animals for Experimental and Other Scientific Purposes. Blood samples were collected, mixed with heparin, and centrifuged at 11.000 ×g for 10 min at 4°C and plasma isolated. Kidneys were harvested, divided in two and either snap-frozen, fixed in a Zn-based fixative (ZBF) (36.7 mmol/L ZnCl_2_, 27.3 mmol/L ZnAc_2_ × 2H_2_O and 0.63 mmol/L CaAc_2_ in 0.1 mmol/L Tris pH 7.4), or fixed in 2% (w/v) glutaraldehyde, 0.5% (w/v) paraformaldehyde in 0.1 mol/l phosphate buffer, pH 7.4. The animal experiments were approved by the Animal Welfare Committee, University of Tromsø.

### 2.2. Zymography

Presence of gelatinolytic enzymes in plasma and kidney extracts from the mice were analyzed by SDS-PAGE gelatin zymography as previously described in detail [24]. After incubations, the gels were stained with 0.2% (w/v) coomassie brilliant Blue R-250. Gelatinolytic activity was evident as cleared regions on the gel. Positive controls for MMP-9 and MMP-2 were as described [25]. Parallel gels were run with 0.1% (w/v) plasminogen (Glu-type, from human plasma), in addition to gelatin to assess the presence of plasminogen activators in samples. To further identify enzymes responsible for gelatinolytic activity, parallel gels were run with inhibitors added in the washing and incubation buffers. Inhibitors were either the metalloproteinase inhibitor EDTA (10 mmol/L), the serine proteinase inhibitor Pefabloc (1 mmol/L), or TLCK (Na-tosyl-Lys-chloromethylketone) (1mmol/L) which inhibits trypsin and trypsin-like enzymes, but not chymotrypsin.

### 2.3. Western Blotting

Plasma fractions obtained after gelatin affinity chromatography were electrophoresed on SDS-PAGE with 4% polyacrylamide (w/v) in stacking gel and 7.5% (w/v) in separating gel and electroblotted to a polyvinylidene difluoride membrane (Millipore, Billerica, MA, USA). After blockage of nonspecific binding sites with 5% (w/v) non fat milk in Tris-buffered saline, blots were incubated for 1 h at room temperature with antibodies against human MMP-2 (R&D systems, Minneapolis, MN, USA) and MMP-9 (Chemicon/Millipore, Billerica, MS, USA). After washing, the blots were incubated for 1 h at room temperature with horseradish peroxidise- (HRP-) conjugated antibodies MMP-2: Anti-goat (Santa Cruz, CA, USA), MMP-9: Anti-rabbit (Southern Biotech, Birmingham, AL, USA) in blockage solution and developed with Western Blot Luminol (Santa Cruz).

### 2.4. Electron Microscopy

Kidneys were divided in two, and one part was fixed in 2% (w/v) glutaraldehyde (Agar Scientific, Stansted, Essex, UK), 0.5% (w/v) paraformaldehyde (Chemi-Teknikk, Oslo, Norway) in 0.1 mol/L phosphate buffer, pH 7.4. Four samples of cortical tissue from each of these half kidneys were cut out, postfixed in 2% (w/v) osmium tetroxide (Agar Scientific), dehydrated in graded alcohol, and embedded in epoxy resin (Agar Scientific). Semithin sections were cut from coded blocks, stained with toluidine blue, and used for light microscopic morphometry using semiautomatic interactive image analysis (AnalySIS 3.0, Soft Imaging Systems, Münster, Germany). Thus, from each of 8 animals, the glomerular surface area was measured for all glomerular profiles of one section from each of 6 independent cortical tissue blocks, and the 6 largest profiles were subjected to further analyses. One block containing central sections of 2 glomeruli was chosen for ultrathin sectioning followed by uranyl acetate/lead citrate contrasting. At medium power electron microscopy, the sections of the glomerular tufts were scanned for thin capillary loops. Five such loops were subjected to high-power microscopy, and the thickness of the basement membrane measured from the 5 thinnest areas of each loop on digital images using semiautomatic interactive image analysis as above. Data from the two animal groups were analyzed for possible statistical differences using student *t*-test. *P* values <0.05 were considered significant.

### 2.5. Immunohistochemistry

Presence and localization of MMP-2 and MMP-9 in kidneys from 4 parallel db/db and db/+ mice was studied by immunohistochemistry using polyclonal antibodies against MMP-2 and MMP-9 (Abcam, Cambridge, MA, USA). Four-*μ*m thick sections of ZBF-fixed, paraffin-embedded kidneys on Superfrost Plus slides were deparaffinised in xylene and rehydrated in graded alcohol baths. Immunohistochemistry was performed as previously described [24]. In brief, primary antibodies (MMP-2 and MMP-9) were incubated 30 min at room temperature after blocking of endogenous peroxidase activity and unspecific binding. Thereafter, HRP-labelled secondary antibody was incubated 30 min and visualized with a DAB substrate (EnVision^+^ system-HRP for rabbit primary antibodies, DAKO (Glostrup, Denmark)). Nuclei were counterstained with Harris haematoxylin (Chemi-Teknikk) and sections mounted with Histokit (Chemi-Teknikk). As negative control, the primary antibody was replaced by 1.5% normal goat serum (Dako) in some sections.

### 2.6. In Situ Zymography

For localization of gelatinolytic activity, in situ zymography was performed as previously described [24] on the same kidneys as used for immunohistochemistry. Briefly, 5 *μ*m thick sections of ZBF-fixed, paraffin-embedded kidneys were deparaffinised in xylene and rehydrated in graded alcohol baths. 1 mg DQ-gelatin substrate (Invitrogen, Carlsbad, CA, USA) was dissolved in 1.0 mL Milli-Q water and diluted 1 : 50 in 50 mmol/L Tris-HCl, 150 mmol/L NaCl, 5 mmol/L CaCl_2_, and 0.2 mmol/L sodium azide (pH 7.6). Sections were incubated with 250 *μ*L of this mixture in a dark humidity chamber at 37°C for 2 h, rinsed, and fixed in 4% (w/v) neutral-buffered formalin. Nuclei were counterstained with DAPI (Invitrogen). To verify the contribution of metallo- and serine proteinases, control slides were preincubated with 20 mmol/L EDTA alone or with 1 mmol/L Pefabloc for 1 h. The inhibitors were also added to the substrate. The level of autofluorescence in the tissue was evaluated by incubating control sections at −20°C for 2 h immediately after the substrate was added. Fluorescence was studied using an Olympus BX51 fluorescence microscope with Cell^F^ imaging software (Olympus, Tokyo, Japan).

### 2.7. Extraction of Proteinases from Kidneys

Unfixed kidneys from 3 parallel db/db and db/+ mice were homogenized with 20 *μ*L of lysis buffer (0.25% (v/v) Triton X-100 and 10 mmol/L CaCl_2_ in 0.1 mol/L Hepes, pH 7) per mg tissue (wet weight), using a Tissue Lyser (Qiagen, Hilden, Germany) with 5 mm steal beads at 25 Hz, 2.5 minutes × 2 (4°C) [24]. The tubes were then centrifuged at 10.000 ×g for 5 min (4°C) and the supernatants harvested.

## 3. Results

### 3.1. Gelatin-Degrading Proteinases in Mouse Plasma

**Figure 1:**
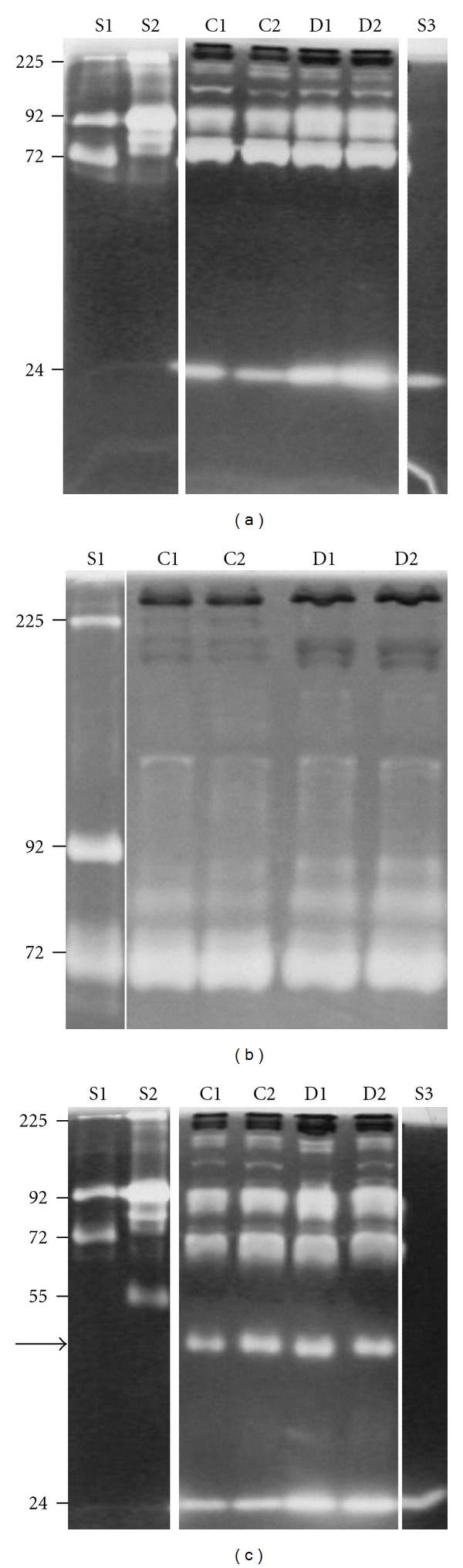
SDS-PAGE gelatin and plasminogen zymography of plasma from db/db and db/+ mice. Plasma from db/+ (C1 and C2) and db/db (D1 and D2) mice was subjected to gelatin zymography with 10% polyacrylamide in the gels (a). For better separation in the higher molecular weight areas, the samples were also run on 7.5% polyacrylamide gels (b). The same samples were further subjected to gelatin and plasminogen zymography to detect both gelatinases and plasminogen activators (c). Standards used were S1 with pro-MMP-2, (72000), pro-MMP-9 monomer (92000), and pro-MMP-9 homodimer (225000), S2 (conditioned medium from the monocytic cell line THP-1) containing pro-MMP-9 monomer (92000) and pro-MMP-9 homodimer (225000), and plasminogen activator (55000) and S3 containing trypsin (24000). The gels were stained with Coomassie blue, and white areas indicate gelatinases. The arrow in panel C shows the position of a plasminogen activator in mouse plasma samples. These zymograms are representative for all the 20 plasma samples investigated.

Plasma from both diabetic (db/db) and control mice (db/+) contained gelatinolytic enzymes (Figure 1(a)). The most prominent gelatinases had* M_r_* of approximately 24000, 72000, and 92000, but enzymes with* M_r_* above 92000 were also detected. The gelatinase bands at* M_r_* 24000 and 92000 were more prominent in plasma from db/db mice than from the control db/+ mice, whereas all other bands were similar in plasma from the two different animal groups. The difference in gelatin-degrading enzymes in samples from db/db and db/+ mice, as determined by SDS-PAGE gelatin zymography using 10% (w/v) polyacrylamide, was further analyzed by using a 7.5% (w/v) polyacrylamide gel to obtain better separation of enzymes with* M_r_* between 72000 and 92000. From this experiment (Figure 1(b)), it is evident that the gelatinases detected with* M_r_* of approximately 92000 (Figure 1(a)) were derived from two distinct bands with approximate* M_r_* of 80000 and 85000. No difference was observed in the band with* M_r_* of 72000. These results show that plasma from db/db mice contained more gelatin-degrading enzymes with* M_r_* of 24000, 80000, and 85000 compared to samples from db/+ mice. 

 To further analyze for the presence of plasminogen activators in plasma from control and diabetic mice, samples were subjected to zymography with gels containing both gelatin and plasminogen substrates. In Figure 1(c), the same gelatinases as those shown in Figure 1(a) can be observed. In addition, a band with* M_r_* of approximately 45000 (arrow) was observed in all four samples but with the same amounts in diabetic and control mice.

### 3.2. Nature of the Gelatin-Degrading Proteinases in Mouse Plasma

**Figure 2:**
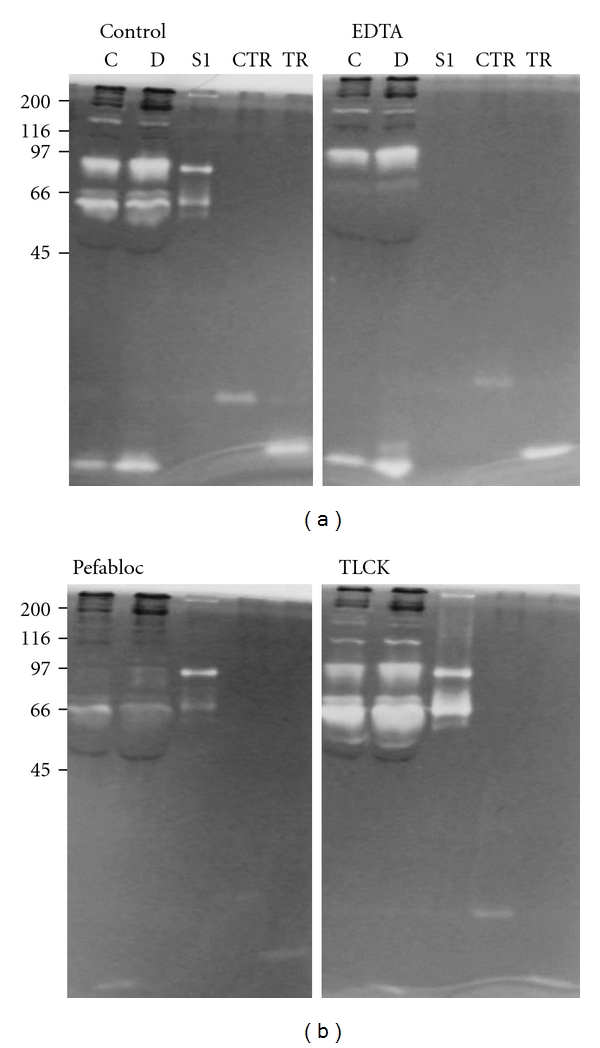
SDS-PAGE gelatin zymography with different inhibitors. Plasma from db/+ (C) and db/db (D) mice was subjected to gelatin zymography with 10% polyacrylamide in the gels without treatment (Control), after incubation with 10 mmol/L EDTA (EDTA), 1 mmol/L Pefabloc (Pefabloc) or 1 mmol/L TLCK. Standards used were S1 as in Figure 1, chymotrypsin (CTR), and trypsin (TR). Shown at the left is the molecular size of protein *M_r_* markers.

To further identify the gelatinases found in plasma from db/db mice and db/+ mice, samples were subjected to zymography in the absence and presence of different inhibitors. When the samples were incubated with EDTA, the gelatinases with* M_r_* of 80000–850000 and 24000 were not affected, whereas the gelatinase with *M_r_* of 72000 was completely abolished (see Figure 2 panel labelled EDTA). Protein standards were run under reducing conditions while the plasma samples were run under nonreducing conditions, which explains why the 72000 pro-MMP-2 standard has an* M_r_* slightly less than 66000. The gelatinase with* M_r_* of 72000 found in both type of samples is, accordingly, a metalloproteinase. Plasma samples were also treated with the serine proteinase inhibitor Pefabloc, which completely abolished all gelatinase activities except the gelatinase with * M_r_* of 72000 (Figure 2, panel labelled Pefabloc), demonstrating that the gelatin-degrading enzymes with* M_r_* of 24000, 80000, and 85000 in samples from both db/db and db/+ mice are serine proteinases. Finally, we also used TLCK, which is an inhibitor of trypsin and trypsin-like enzymes but not of chymotrypsin. This treatment inhibited only the gelatin-degrading enzyme with* M_r_* of 24000 (Figure 2, panel labelled TLCK). In summary, the amounts of serine proteinases with* M_r_* of 24000, 80000, and 85000 are higher in plasma samples from db/db mice compared to db/+ control mice, and the enzyme with* M_r_* of 24000 is either mouse trypsin or a trypsin-like serine proteinase. 

**Figure 3:**
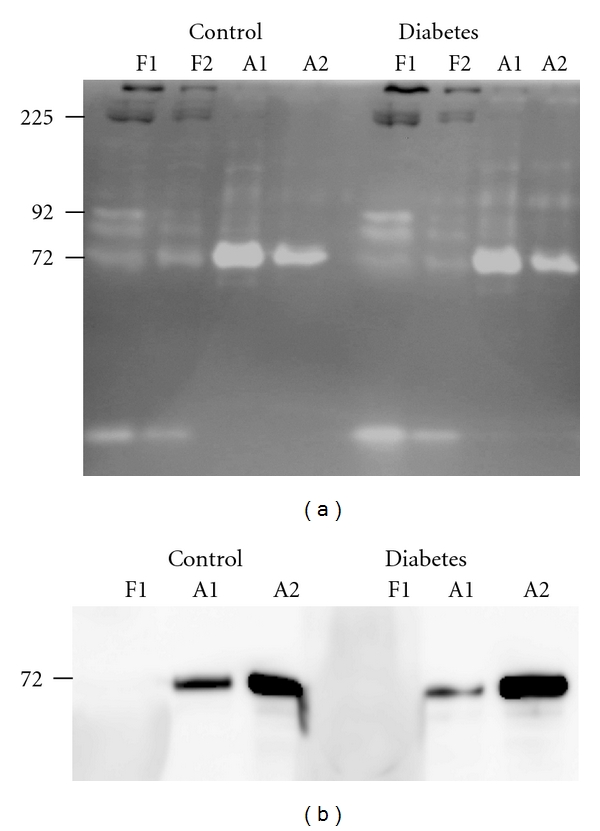
SDS-PAGE gelatin zymography and Western blotting after gelatin affinity chromatography. Plasma samples from db/+ (Control) and db/db (Diabetes) mice were subjected to gelatin affinity chromatography followed by SDS-PAGE gelatin zymography (a) and Western blotting (b). Western blotting was performed with an antibody against MMP-2 on the flow through fraction (F1) and the fractions eluted with 5% DMSO (A1 and A2) from the affinity column. The migration of standard S1 (as in Figure 1) and the pro-MMP-2 standard is shown at the left of panel A and B, respectively.

 Some of the gelatin-degrading proteinases found in plasma samples from control and diabetic mice could belong to the MMP family. To test this, we performed Western blotting experiments using antibodies against MMP-2 and MMP-9. In the initial experiments, we could not detect these MMPs with the antibodies used (not shown). Therefore, prior to Western blotting, plasma samples were subjected to gelatin affinity chromatography to remove components that could potentially interfere with Western blotting. Gelatin zymography (Figure 3(a)) showed that the gelatin-degrading enzyme with* M_r_* of 72000 bound to the gelatin-Sepharose column (A1 and A2), while the enzyme with* M_r_* of 24000 and two species with* M_r_* of approximately 92000 passed through (F1 and F2). No MMP-9 was detected in the Western blots (not shown). However, MMP-2 was detected in the fractions bound to the column (A1 and A2) as shown in Figure 3(b). Thus, the metalloproteinase in plasma with* M_r_* of 72000 from both control and diabetes mice detected by gelatin zymography is MMP-2.

### 3.3. Morphometry

**Figure 4:**
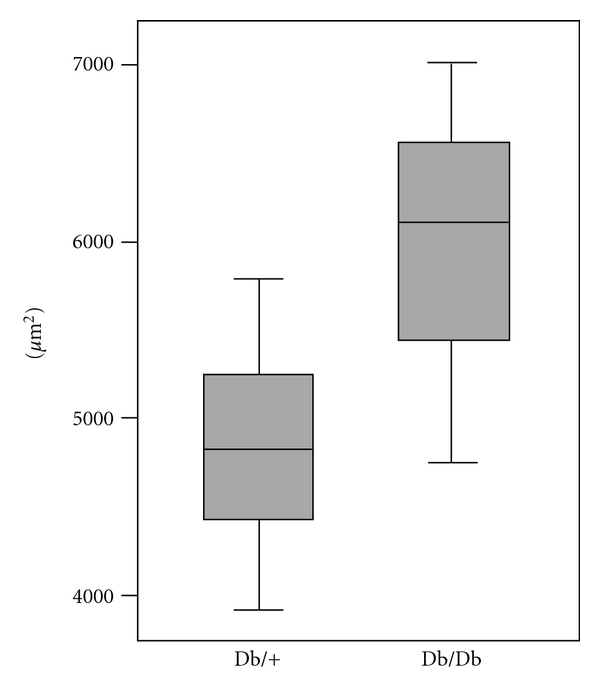
Glomerular surface area of kidney biopsies from db/db and db/+ mice. Sections of epoxy resin-embedded kidney cortical samples from db/db and db/+ mice were subjected to light microscopic morphometry by interactive image analysis. Kidney samples were obtained from 8 animals in each group, and 6 sections were analyzed from each animal. Box plot shows median glomerular surface area (*μ*m^2^).

To document morphological changes in the sections of cortical kidney, tissue from eight db/db and eight db/+ mice were subjected to light microscopic morphometry. The results showed larger glomerular surface areas in sections from db/db mice than in corresponding sections from db+ mice (Figure 4). The difference between the two groups was statistically significant (*P* = 0.004). At the ultrastructural level, morphometry was performed to measure basement membrane thickness in glomerular capillary loops. There was a tendency towards thicker basement membranes in glomeruli of db/db mice compared to controls (Table 1), although the difference was not statistically significant (*P* = 0.27). The control material contained one outlier (no. 21). If this was excluded, the difference between the two groups was statistically significant (*P* = 0.033).

### 3.4. Immunohistochemistry

**Figure 5:**
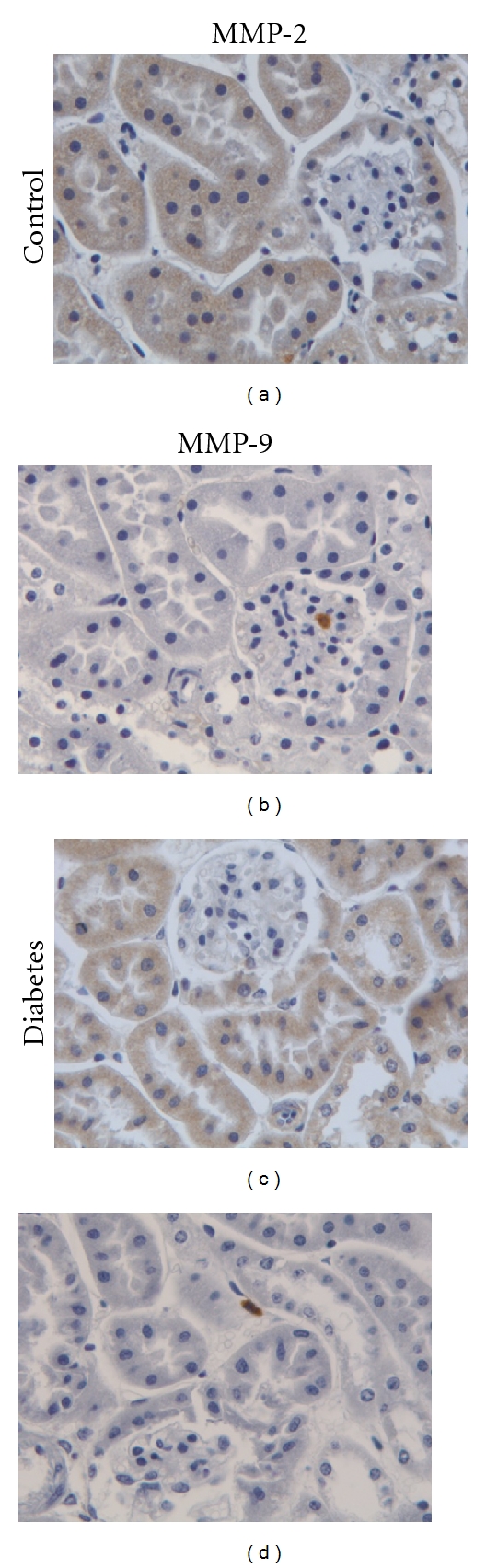
Immunohistochemistry of kidney tissue from db/db and db/+ mice. Kidney sections from db/+ mice (Control) and db/db mice (Diabetes) were subjected to immunohistochemistry using antibodies against MMP-2 (left panels) and MMP-9 (right panels). Magnification is 400x.

The kidney samples were also subjected to immunohistochemistry to test whether MMP-2 and MMP-9 were differently expressed in the db/db and db/+ mice. MMP-2 could be detected both in glomeruli and the tubule systems of kidneys from control and diabetes mice (Figure 5). However, no striking difference in staining intensity could be observed between samples from db/db and db/+ mice. With the MMP-9 antibody, prominent staining was seen in a few scattered cells, otherwise only weak staining was observed in kidneys from both db/db and db/+ mice.

### 3.5. In Situ Zymography

**Figure 6:**
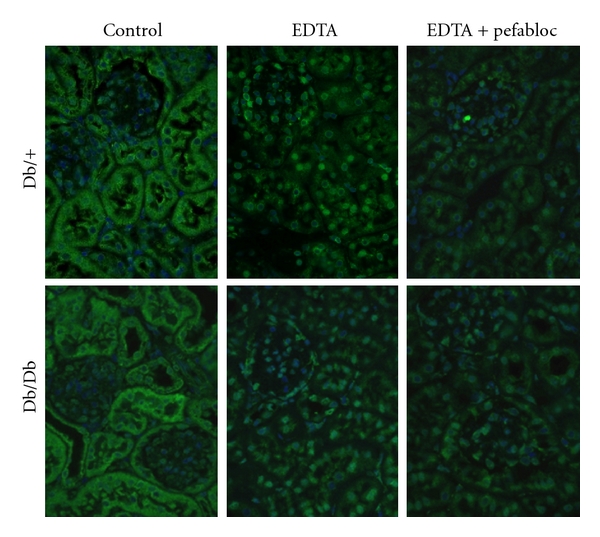
In situ zymography of kidneys from db/db and db/+ mice. Kidney sections from db/+ mice (Db/+) and db/db mice (Db/Db) were subjected to in situ zymography. Sections were either incubated without inhibitors (Control), in the presence of 20 mmol/L EDTA (EDTA), or with both 20 mmol/L EDTA and 1 mmol/L Pefabloc (EDTA + Pefabloc). Areas with gelatinolytic activity are shown as green fluorescence. Nuclei were counterstained with DAPI. Magnification is 400x.

To further analyze for possible presence and activity of gelatin-degrading enzymes in the kidneys, sections from db/db and db/+ mice were subjected to in situ zymography in the absence and presence of EDTA and Pefabloc. Gelatinolytic activity was primarily detected in the tubule system in both control and diabetic mice (Figure 6, panel Control). When parallel sections were incubated with EDTA, a large decrease in gelatinolytic activity was evident (panel EDTA). Finally, a further decrease was observed with both EDTA and Pefabloc, demonstrating the presence of gelatinolytic activities of both metalloproteinase and serine proteinase nature in the kidney tissue. However, no difference in gelatinase activities could be detected between kidney specimens from db/db and db/+ mice.

### 3.6. Gelatin-Degrading Proteinases in Kidney Extracts

**Figure 7:**
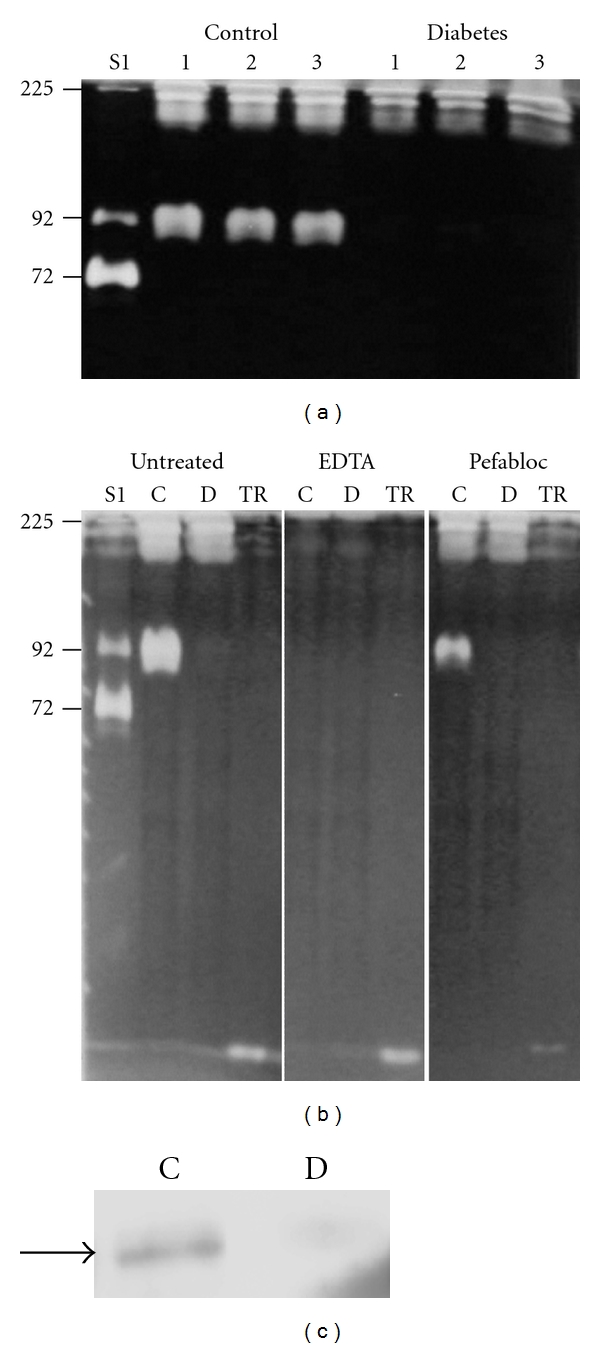
Gelatin zymography and Western blotting of kidney extracts from db/db and db/+ mice. (a) kidney extracts from three different db/+ mice (Control) and db/db mice (Diabetes) were subjected to zymography. The molecular size standards used are the same as those described in the legend to Figure 1. (b) extracts from db/+ (C) and db/db (D) mice were also subjected to gelatin zymography with or without EDTA (10 mM) or Pefabloc (1 mmol/L) in the washing and incubation buffers. Molecular size standards used were as described in the legend to Figure 1. TR: Trypsin. (c) extracts from db/+ (Control) and db/db (Diabetes) mice were subjected to lentil lectin-Sepharose chromatography followed by Western blotting using an antibody against MMP-9. The arrow shows the migration of the pro-MMP-9 (92000) standard.

To further address the question if changes in gelatin-degrading proteinases in plasma in db/db mice are related to similar changes in the kidneys, extracts of kidney tissues were prepared and analyzed by gelatin zymography. Both extracts contained high molecular weight gelatin-degrading proteinases (*M_r_* around 225000), but proteinases migrating as the pro-MMP-9 standard were only present in extracts from control db/+ mice, as shown in Figure 7. In striking contrast to the observations on plasma samples (see Figure 1), no such proteinases could be detected in kidney extracts from db/db mice (Figure 7). Gelatin zymography was further performed in the absence and presence of either EDTA or Pefabloc. The gelatin-degrading proteinases migrating as the pro-MMP-9 standard were completely abolished with EDTA (Figure 7(b)) but not with Pefabloc treatment. In conclusion, kidney extracts of db/+ mice contain a gelatin-degrading enzyme with* M_r_* of 92000 which was of metalloproteinase nature. This enzyme was completely absent in extracts of corresponding tissues from the db/db mice. 

 To further investigate if this metalloproteinase was MMP-9, kidney extracts were subjected to Western blotting. In the initial experiments, we could not detect MMP-9 (not shown). Extracts were, therefore, subjected to both gelatin-Sepharose and lentil lectin-Sepharose affinity chromatography. MMP-9 is known to bind gelatin through their fibronectin repeats [26] and to lentil lectin through their glycan chains [27, 28]. Interestingly, the enzyme with* M_r_* of 92000 from the kidney extract did not bind to the gelatin-Sepharose column but to the lentil lectin column (not shown). Western blotting revealed that at least a part of the enzyme from the db/+ mice extracts with* M_r_* of 92000 binding to the lentil lectin column was MMP-9, as can be seen in Figure 7(c). No MMP-9 could be detected in corresponding material from db/db mice (Figure 7(c)).

## 4. Discussion

In the present study, we have analyzed gelatinolytic enzymes in plasma and kidney samples from db/db mice and corresponding db/+ control mice. The results presented show that plasma from db/db mice contains increased amounts of gelatin-degrading serine proteinases compared to controls. Further, kidney extracts of db/db mice showed a complete loss of gelatinases with* M_r_* of 92000 compared to extracts from the control mice. In addition, in situ zymography demonstrated that metallo- and serine proteinase activity was mainly found in the tubular system. We also show that the glomerular surface area is larger in db/db mice than in db/+ mice. Our data do also indicate an increased thickness of glomerular basement membranes in db/db mice, in line with other observations from db/db mice [8].

 The use of well-suited animal models with relevance for human diabetes and kidney complications, in particular, is an important issue. The generation of disease markers, further understanding of pathological mechanisms, testing and generation of new treatments, all depend on good and relevant animal models. The db/db mouse is one such model, in addition to, for example, the ob/ob mouse, the KKAy mouse, the New Zealand obese mouse, and the streptozotocin-induced type 1 diabetes mouse. For studies of kidney changes, the db/db mouse may be a good model system [23]. Rat has also been suggested as a well-suited model [22]. Some studies in streptozotocin-induced diabetic rats have shown decreased MMP-9 activity in the kidneys [29, 30]. However, increased MMP-2 staining in the kidneys of diabetic rats has also been demonstrated, as well as an imbalance between MMP-2 and TIMP-2 but with no data reported regarding changes in MMP-9 expression [17]. Finally, in one of the limited number of studies in mice, an increase in MMP-9 was observed in the KKay mouse model, in contrast to what our study shows. Clearly, there is need for more studies in mouse and other animal models, with relevance for diabetes-induced kidney changes in humans with diabetes. In addition, the issue of early markers in plasma for predicting kidney changes in diabetes, as has been addressed in the present study, needs to be expanded in future studies. Such markers can also be useful in studies evaluating different treatments for kidney complications, as has been done to a certain extent [29–31]. 

 The data presented show a major decrease in a gelatinase with* M_r_* of 92000, (consistent with MMP-9) in kidney extracts from db/db mice, which is not reflected in blood samples. Changes in ECM turnover may, therefore, be more prominent in some tissues than others in diabetes. This is an important issue in diabetes research because several organs are affected by the diabetic state. The question is if there is a hyperglycaemia and time dependence of organ affections and to what extent blood markers can be used to predict potential complications in different organs at risk.

 The data presented here using plasma from db/db mice did not show the same changes in gelatinases as we observed in a previous study on type 1 diabetes in humans. Blood samples from patients with type 1 diabetes contained higher concentrations of MMP-2 and MMP-9 compared to healthy controls [20]. Increased plasma MMP-2 and MMP-9 has also been demonstrated in persons with type 2 diabetes and peripheral arterial disease [32]. Increased amount of gelatinolytic enzymes were observed in the db/db mouse blood samples compared to control samples. Interestingly, the elevated amount of enzymes was shown to be serine proteinases and not MMPs. The identification of these proteinases is the subject of further studies. 

 The kidney extracts from db/+ control mice contained a metalloproteinase with* M_r_* of 92000 which was not found in extracts from db/db mice. Western blotting revealed that at least part of this band corresponded to MMP-9. MMP-9 and MMP-2 are known to degrade ECM components such as collagen type IV [33, 34]. Our EM data show that the glomerular surface area increased and that basement membranes from kidneys of db/db mice were thicker than those of db/+ mice. A major part of the ECM accumulation in db/db mice is due to increased concentration of collagen type IV, fibronectin, and laminin [23]. The decrease in the gelatinase with* M_r_* of 92000 in kidney extracts from db/db mice may contribute to such ECM accumulation. The dynamics of ECM turnover is clearly affected in the kidneys of db/db mice contributing to albuminuria and kidney changes [23], as also observed in humans [14]. By in situ zymography, we demonstrated that metalloproteinases were largely confined to the tubular system. If changes in filtration properties in the diabetic kidney are linked to changes in ECM and metalloproteinases, in particular, these data point to the importance of focusing on such enzymes in kidney tubules in future studies. 

 In human diabetes, it is well documented that the mesangial matrix is expanded and that the basement membranes of glomeruli are thicker than controls [2]. The molecular basis for such changes is not well defined, but a multitude of hypotheses have been discussed. Several studies have indicated that important issues to address are proteinase/inhibitor systems, profibrotic cytokines, and several ECM components [8, 35]. In kidneys of db/db mice, an increased accumulation of ECM, linked to increased expression of TGF-*β*, has been established [36]. Experimental studies have shown that inhibiting TGF-*β* signalling in db/db mice reversed glomerulopathy and mesangial matrix expansion [37, 38]. The results from the present study suggest that serine proteinases and metalloproteinases should also be addressed in more depth as potential targets for experimental treatment of kidney complications in diabetes. The db/db mouse would be a useful model for such experiments. Furthermore, it would be relevant to address whether changes in circulating serine proteinases and metalloproteinases in kidneys are related to how well the blood glucose level is regulated. Previous studies, using human kidney biopsies from patients with type 1 diabetes, have demonstrated that increased thickness of glomeruli basement membrane in biopsies can be reversed if blood glucose levels are tightly controlled [39, 40].

## 5. Conclusions

Plasma from db/db mice contained larger amounts of serine proteinases compared to db/+ mice indicating that levels of serine proteinases in plasma may serve as a marker of kidney changes in db/db mice. Furthermore, kidney extracts from db/+ mice contained more MMP-9 than corresponding extracts from db/db mice. This decrease in MMP-9 might contribute to the altered tissue homeostasis in the kidneys of db/db mice, as demonstrated by increased glomerular surface area and thicker glomerular basement membranes, compared to the db/+ mice. There are several issues that need to be addressed in further studies on serine proteinases and MMPs in relation to diabetes and kidney complications. The actual targets for MMPs are poorly defined in kidneys, and the possible importance of MMP changes for diabetes-related pathogenesis and ECM alterations has so far not been addressed in any detail. There is a potential to use MMPs and related proteinases in plasma as markers for kidney changes. MMPs and TIMPs may also be potential targets for therapeutic interventions. The db/db mouse model is useful for studying kidney changes in diabetes [23], and our data suggests that further studies should address the regulation of proteinases in both plasma and kidneys in more detail.

## Figures and Tables

**Table 1:** Basement membrane thickness of glomeruli from kidney biopsies of db/db and db/+ mice.

Diabetes (db/db) Animal no.	Basement membrane thickness (nm) with (SD)	Control (db/+) Animal no.	Basement membrane thickness (nm) with (SD)
1	116,8 (5,9)	11	125,3 (0,7)
2	124,7 (3,2)	12	117,8 (0,4)
3	120,6 (2,0)	13	116,2 (2,5)
4	126,3 (2,7)	14	109,1 (3,5)
5	119,7 (1,0)	15	107,8 (2,4)
6	117,8 (4,8)	16	107,3 (1,2)
7	105,3 (1,8)	17	104,6 (0,5)
8	110,1 (0,7)	18	107,1 (1,6)
		19	105,2 (0,3)
		20	107,0 (0,9)
		21	135,2 (9,1)
		22	107,2 (2,0)
Mean (SD)	117,7 (7,0)		112,5 (9,5)
